# A Longitudinal Electromyography Study of Complex Movements in Poststroke Therapy. 1: Heterogeneous Changes Despite Consistent Improvements in Clinical Assessments

**DOI:** 10.3389/fneur.2017.00340

**Published:** 2017-07-28

**Authors:** Negin Hesam-Shariati, Terry Trinh, Angelica G. Thompson-Butel, Christine T. Shiner, Penelope A. McNulty

**Affiliations:** ^1^Neuroscience Research Australia, Sydney, NSW, Australia; ^2^School of Medical Science, University of New South Wales, Sydney, NSW, Australia

**Keywords:** muscle activation, movement duration, motor-function, upper limb, rehabilitation, chronic stroke

## Abstract

Poststroke weakness on the more-affected side may arise from reduced corticospinal drive, disuse muscle atrophy, spasticity, and abnormal coordination. This study investigated changes in muscle activation patterns to understand therapy-induced improvements in motor-function in chronic stroke compared to clinical assessments and to identify the effect of motor-function level on muscle activation changes. Electromyography (EMG) was recorded from five upper limb muscles on the more-affected side of 24 patients during early and late therapy sessions of an intensive 14-day program of Wii-based Movement Therapy (WMT) and for a subset of 13 patients at 6-month follow-up. Patients were classified according to residual voluntary motor capacity with low, moderate, or high motor-function levels. The area under the curve was calculated from EMG amplitude and movement duration. Clinical assessments of upper limb motor-function pre- and post-therapy included the Wolf Motor Function Test, Fugl-Meyer Assessment and Motor Activity Log Quality of Movement scale. Clinical assessments improved over time (*p* < 0.01) with an effect of motor-function level (*p* < 0.001). The pattern of EMG change by late therapy was complex and variable, with differences between patients with low compared to moderate or high motor-function levels. The area under the curve (*p* = 0.028) and peak amplitude (*p* = 0.043) during Wii-tennis backhand increased for patients with low motor-function, whereas EMG decreased for patients with moderate and high motor-function levels. The reductions included movement duration during Wii-golf (*p* = 0.048, moderate; *p* = 0.026, high) and Wii-tennis backhand (*p* = 0.046, moderate; *p* = 0.023, high) and forehand (*p* = 0.009, high) and the area under the curve during Wii-golf (*p* = 0.018, moderate) and Wii-baseball (*p* = 0.036, moderate). For the pooled data over time, there was an effect of motor-function (*p* = 0.016) and an interaction between time and motor-function (*p* = 0.009) for Wii-golf movement duration. Wii-baseball movement duration decreased as a function of time (*p* = 0.022). There was an effect on Wii-tennis forehand duration for time (*p* = 0.002), an interaction of time and motor-function (*p* = 0.005) and an effect of motor-function level on the area under the curve (*p* = 0.034) for Wii-golf. This study demonstrated different patterns of EMG changes according to residual voluntary motor-function levels, despite heterogeneity within each level that was not evident following clinical assessments alone. Thus, rehabilitation efficacy might be underestimated by analyses of pooled data.

## Introduction

Motor impairment is the most common outcome after stroke (1–4) and is predominately attributed to muscle weakness (5–7) as a consequence of reduced corticospinal drive (8), disuse muscle atrophy (9, 10), impaired voluntary control of muscles (11, 12), spasticity (13–16), and impaired muscle coordination (17–19). These factors, either in isolation or combination, result in abnormal muscle activation during voluntary movements (20–22). Multifactorial contributions to impaired upper limb motor-function are more common than in the lower limb (23, 24) and a more important focus for improving independence in activities of daily living (25, 26). Poststroke upper limb recovery may be slower and more complicated than that of the lower limb, given that upper limb tasks are typically more complex involving more degrees of freedom in multi-joint movements (27, 28).

Recovery after stroke is a complex combination of spontaneous neurological mechanisms and relearning processes (28–30). It is commonly thought that learning-dependant mechanisms are only operative during natural recovery and interact with therapeutic interventions (31, 32). Furthermore, true recovery is thought to be complete between 4 and 10 weeks poststroke (31, 33) or reaches a plateau over 6 months (30–32, 34). It has been speculated that any improvement in the chronic period is not true improvement, but rather a restitution of therapy gains made earlier and lost over time (35). Despite this, significant improvements are possible in chronic stroke, but motor-function in this period needs intensive rehabilitation for continued improvements (1, 22, 36).

Clinical motor assessment both after stroke and with rehabilitation is traditionally based on task completion with limited assessment of movement quality (26, 37–39). Most assessments are qualitative with subjective and categorical scoring and many suffer from ceiling and floor effects (40). To provide more objective and quantitative measures, recent studies have examined muscle activation and joint kinematics to evaluate poststroke motor outcomes; yet, most upper limb studies consist of simple tracking tasks (41–43) or reaching movements (27, 34, 44, 45) constrained in time and space.

In this study, electromyography (EMG) analysis was used in addition to clinical assessments to provide quantitative measures of outcomes with therapy in chronic stroke. The aim of this study was to examine changes in muscle activation (EMG) with an intensive 14-day program of Wii-based Movement Therapy Wii-based Movement Therapy to investigate the mechanisms underlying therapy-induced motor improvements. Wii-based Movement Therapy is the equivalent of current best practice in upper limb stroke rehabilitation, Constraint-induced Movement Therapy (46). The stability of motor-function improvements and changes in EMG during therapy were assessed *via* a longitudinal comparison to 6-month follow-up. We hypothesized that there would be distinct patterns of change in EMG and that these would vary according to the level of residual voluntary motor capacity and correlate with improvements in motor-function quantified using clinical assessments.

## Materials and Methods

### Participants

**Figure 1:**
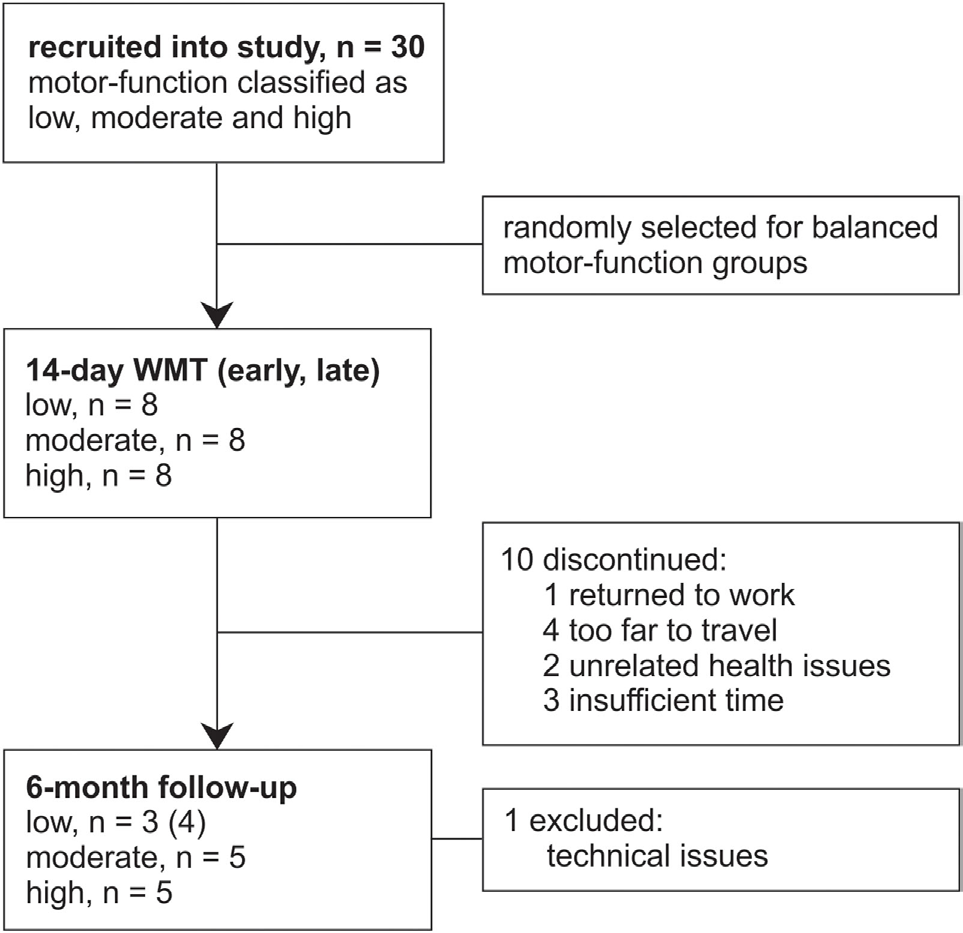
Flow of patients through this study. Patients were recruited from concurrent studies of Wii-based Movement Therapy (WMT) for this data analysis.

The data from 30 patients with chronic stroke (i.e., ≥3 months poststroke) were included in this study from those previously recorded from patients consecutively recruited from St. Vincent’s and Prince of Wales Hospitals, Sydney, for concurrent studies of Wii-based Movement Therapy (Figure 1). The level of residual voluntary motor-function was classified pre-therapy for each patient as low, moderate, or high based on performance of the Box and Block Test of gross manual dexterity and the grooved pegboard test of fine manual dexterity; patients unable to move >1 block in Box and Block Test were classified with low motor-function, those able to move >1 block but unable to complete the pegboard test were classified with moderate motor-function, and those who could complete the pegboard test were classified with high motor-function (47). Thirteen patients were classified with low, nine patients were classified with moderate, and eight patients were classified with high motor-function. To ensure balanced groups, the data for all eight patients with high motor-function were included and the data for eight patients were randomly selected using a computer-generated algorithm from each group of patients with low and moderate motor-function. The data analysis reported here has not been published previously although the clinical assessment data for all patients have been. Twenty patients were included in other Wii-based Movement Therapy trials investigating cardiovascular fitness (48), lower-limb symmetry (25), and motor-function measures (40); 9 patients were included in a randomized-controlled trial comparing Wii-based Movement Therapy and modified Constraint-induced Movement Therapy (46); and 20 patients were also included in a genotype study (49). The inclusion criteria for this study were as follows: unilateral stroke with a contralesional upper limb deficit; ≥3 months poststroke; English communication; ≥10° of voluntary movement in at least one digit of the more-affected hand; age ≥18 years; medically stable; carer available during home practice; and cognitive competency measured as a Mini-Mental State Examination score of ≥24. Exclusion criteria included co-morbidities significantly affecting upper limb sensorimotor function, unstable blood pressure, frail skin that prevented sensor placement and adhesion during recordings, and concurrent formal upper limb therapy. Demographics and baseline characteristics are listed in Table 1. All patients gave signed informed consent to the studies, which were approved by St. Vincent’s Hospital Human Research Ethics Committee, Sydney, and conducted in accordance with the Declaration of Helsinki. All patients were enrolled in a standardized 14-day program of Wii-based Movement Therapy. No attempt was made to control for activities after this period and prior to the 6-month follow-up, although suggestions were made to each patient on how they might best maintain therapy-induced gains. A follow-up session was conducted for all available patients, i.e., a subset of 13 patients at 6-months post-therapy (3 low, 5 moderate, and 5 high motor-function levels). As shown in Figure 1, 10 patients were unavailable, 1 patient returned to work, 4 patients had moved interstate or overseas, 2 patients had unrelated health problems, 3 patients had insufficient time for neurophysiological recordings, and the data for 1 patient was lost due to technical issues.

**Table 1:** Baseline patient characteristics.

Motor-function level	Low	Moderate	High	All	
*N*	8	8	8	24	
Age	59.1 ± 13.9	55.1 ± 11.3	59.4 ± 12.2	57.9 ± 12.1	Range (37–80)
Sex (F/M)	4/4	2/6	2/6	8/16	
More-affected dominant (Y/N)	1/7	3/5	3/5	7/17	
Stroke type (isch/haem)	3/5	7/1	6/2	16/8	
Time poststroke (months)	33.3 ± 9.4	27.3 ± 8.0	19.6 ± 4.5	26.7 ± 4.3	Range (3–88)
Baseline WMFT-tt (s)	81.6 ± 6.9	26.5 ± 10.4	6.3 ± 2.6	38.1 ± 7.8	
Baseline FMA (/66)	25.3 ± 3.4	53.1 ± 3.0	61.6 ± 1.5	46.7 ± 3.6	
Baseline MALQOM (/150)	18.9 ± 11.2	60.0 ± 14.3	101.5 ± 14.7	60.1 ± 8.7	

*Age is reported as mean ± SD, remaining data are reported as mean ± SE. More-affected dominant indicates that the more-affected side is the dominant side*.

*Isch, ischemic; haem, hemorrhage; WMFT-tt, mean time for the Wolf Motor Function-timed tasks where a lower time indicates better motor-function; FMA, upper limb motor Fugl-Meyer Assessment; MALQOM, Motor Activity Log Quality of Movement scale*.

### Therapy

**Figure 2:**
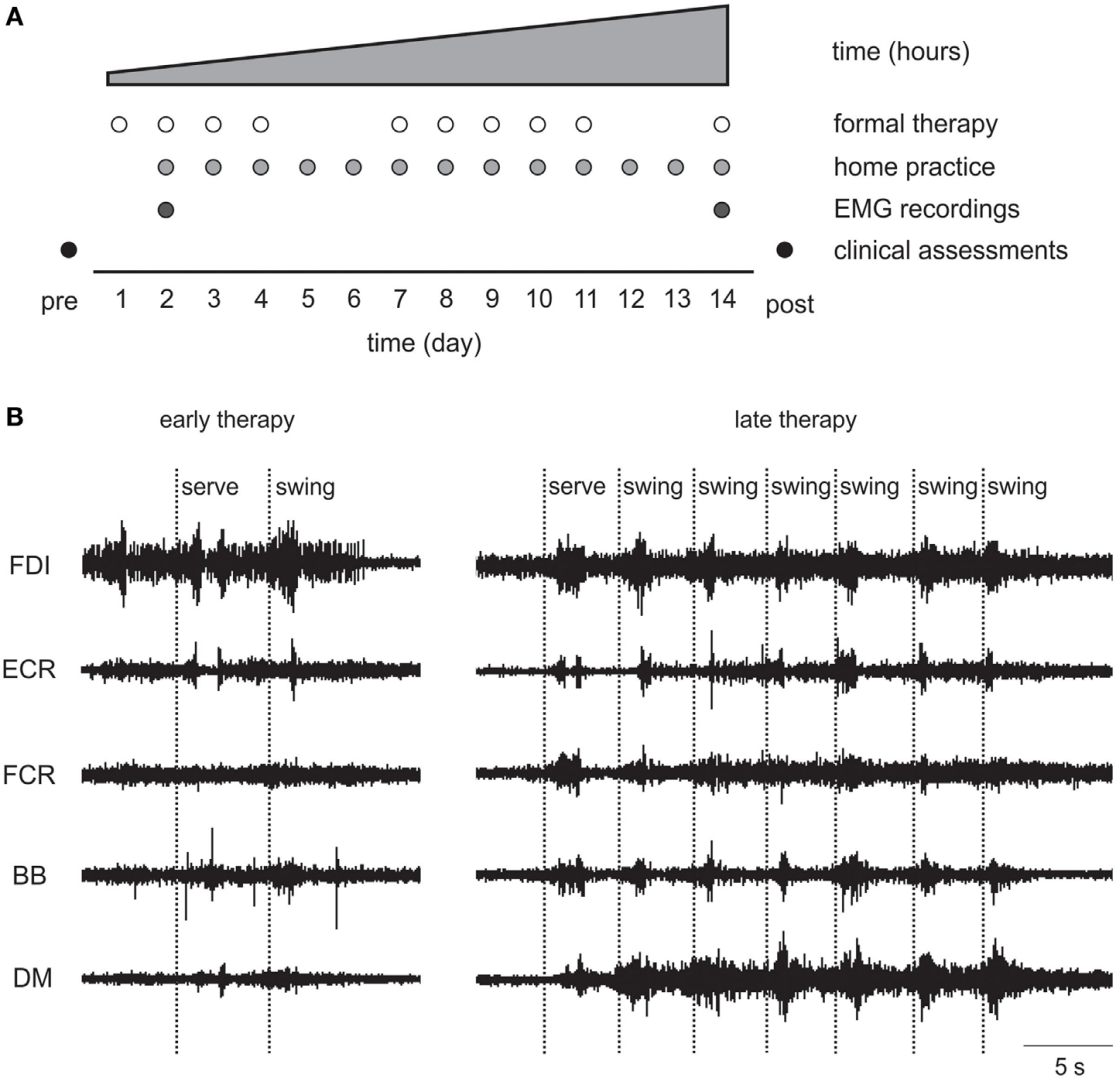
**(A)** Wii-based Movement Therapy (WMT) protocol showing electromyography (EMG) recordings on days 2 and 14. **(B)** Single patient raw EMG at early and late therapy for a 68-year-old female, 46 months poststroke with low motor-function during Wii-tennis. FDI, first dorsal interosseous; ECR, extensor carpi radialis; FCR, flexor carpi radialis; BB, biceps brachii; DM, deltoid medius.

The standardized 14-day Wii-based Movement Therapy program targeted movement quality of the more-affected arm and independence in everyday activities (46, 50). Therapy consists of 1-h formal sessions with an Accredited Exercise Physiologist on 10 consecutive weekdays with increasing prescribed home practice starting on day 2 (see Figure 2A). Wii-based Movement Therapy uses the Nintendo Wii and Wii-Sports games (Nintendo, Japan) of golf, baseball, bowling, tennis, and boxing as a rehabilitation tool in a structured protocol that can be individually tailored to the level of motor-function and progress of each patient (46, 51). Patients used only the more-affected upper limb during therapy activities. When unavoidable, assistance was provided either with the less-affected hand or by the therapist. Game performance was recorded during formal sessions, but the scores were used only for motivational purposes and were not the focus of therapy.

### EMG Recording

Surface EMG data were recorded during formal Wii-based Movement Therapy sessions at two time-points *during* therapy, i.e., at early (day 2–3) and late (between days 12 and 14) therapy; and for a subset of patients at 6-month follow-up. The EMG was not recorded on day 1 to avoid overwhelming patients as they became familiar with the therapy, the device and the therapist. Recordings were made using wireless telemetry sensors (Trigno, Delsys, USA) placed on the following muscles of the more-affected upper limb: deltoid medius (DM), biceps brachii (BB), flexor carpi radialis (FCR), extensor carpi radialis (ECR), and first dorsal interosseous (FDI). Each sensor employs four silver bar electrodes in two pairs with an interelectrode pair distance of 10 mm. Each sensor is optimized for detecting the maximum EMG signal in an orientation perpendicular to the muscle fibers. The small interelectrode distance helps to minimize crosstalk from adjacent muscles. EMG signals were filtered between 20 and 450 Hz, amplified 300 times, sampled at 2 kHz at the source in EMGworks (Delsys, USA), and then analyzed using custom scripts in Spike2 software (CED, UK).

To maximize consistency across patients and sessions, the sensors were placed on the most prominent portion of the muscle belly based on manual palpation during a weak voluntary contraction. Each recording session was preceded by a calibration sequence that consisted of three conditions each held for at least 3 s with the limb segment: (1) supported at rest, (2) unsupported against gravity, and (3) unsupported with the addition of a 1 kg weight placed across the distal joint (51).

### Primary Outcome Measures

Electromyography data were analyzed for movement duration, averaged peak amplitude, and the area under the curve for stereotypical movements of each activity using custom scripts in Spike2 software (CED, UK). Movement duration, peak amplitude, and area under the curve were averaged for 10 consecutive swings of Wii-golf, -baseball, -tennis forehand, and -tennis backhand of each patient at early and late therapy and for a subset of patients at 6-month follow-up. Wii-tennis forehand and backhand swings were analyzed separately due to the need for distinctly different movement patterns. Wii-bowling data were not analyzed as patients with low motor-function could not coordinate the necessary button press, and the speed of Wii-boxing movements particularly for patients with high motor-function was too fast to enable unambiguous movement identification. Therapy was completed standing for all patients except one (moderate) during Wii-tennis at early therapy due to fatigue.

### Secondary Outcome Measures

Upper limb motor-function was assessed using the Wolf Motor Function Test-timed tasks (WMFT-tt) (39) and the upper limb motor Fugl-Meyer Assessment (FMA) (37). The Motor Activity Log Quality of Movement scale (MALQOM) (26) was used to assess independence in activities of daily life. Additionally, the modified Ashworth Scale (52) was used as a clinical measure of muscle resistance at the shoulder, elbow (53), and wrist (54). These clinical tests were measured for all patients immediately pre- (baseline) and post-therapy and for a subset of 13 patients at 6-month follow-up. The clinical assessments were unrelated to the content of therapy.

Although the primary goal of therapy was quality of movement and not game performance, the scores of Wii-golf, -baseball, and -tennis games were recorded during therapy. In Wii-golf, the aim was to successfully land the ball in the hole and each success was noted, while Wii-baseball and -tennis swings were scored when the ball was hit, regardless of whether the hit was successful or not according to the rules of each game.

### Data Analysis

Data analysis for this study was conducted by an independent assessor who was not involved in clinical assessments or therapy delivery. We have used the terms early therapy and late therapy to refer to EMG data recorded during formal therapy sessions and pre- and post-therapy to refer to assessments made using clinical tools prior to and following the therapy protocol.

The EMG data were detrended (DC removed), and root mean square (RMS) processed using a sliding 50 ms window. The mean baseline EMG was measured over 2–3 s prior to the commencement of each activity at rest and then subtracted from the EMG signal. High definition video recordings were used to match the EMG signal to therapy movements. Due to the heterogeneous patterns of motor control and impairment between patients and sports, EMG analysis was targeted to the dominant EMG signal for each patient. Proximal muscles (DM/BB) were primarily analyzed. However, if proximal muscles were silent or tonically active with no phasic activity, and clear task-related activity was evident in a distal muscle (FDI, ECR, or FCR), the distal muscle was used. For example, proximal muscle signals were analyzed in Wii-golf and -tennis except for three and two patients, respectively. In Wii-baseball, the distal muscle signals were more distinct compared to proximal muscles in most patients. For consistency, distal muscle signals were analyzed in Wii-baseball where possible. The same muscle was analyzed for each patient at each time-point.

To identify the onset and offset of therapy movements, a threshold level was set at 5 SD above the mean baseline EMG for each activity of each patient. The movement duration was measured as the interval between the onset and offset of movement; the peak amplitude of each movement was averaged over a 50 ms interval around the absolute peak EMG; and the area under the curve was defined as the area above the baseline level between the onset and offset of the RMS-processed EMG. Both peak amplitude and area under the curve were normalized to the *weighted* condition of the calibration sequence to enable comparison between patients.

### Statistical Analysis

Data were compared for each motor-function group (low, moderate, and high) from early to late therapy (EMG analysis), or pre- to post-therapy (clinical measures) using paired *t*-tests for normally distributed data and reported as mean and SE; otherwise using Wilcoxon signed-rank tests, reported as median and interquartile range.

Longitudinal data were analyzed using mixed models of repeated measures with factors of motor-function (low, moderate, and high) and time (pre-therapy/early therapy, post-therapy/late therapy, and 6-month follow-up). Linear mixed models provide unbiased estimates for the missing data (55) at 6-month follow-up and are more powerful and flexible (56, 57) than repeated measures ANOVA in the presence of multiple missing data points because of the emphasis on the pattern of change rather than the quantitative difference (57).

The relationship between the change in area under the curve and clinical assessments was investigated using Spearman’s rank-order correlation with Bonferroni corrections for multiple comparisons. Statistical analyses were conducted in SPSS 23 software (IBM, USA), and differences were considered significant when *p* < 0.05.

## Results

All patients completed all formal therapy sessions, home practice, and clinical assessments; 10 patients were unavailable for 6-month follow-up (Figure 1). Data are reported at early and late therapy for Wii-golf putting (*n* = 23), -baseball swing (*n* = 24), and -tennis forehand and backhand (*n* = 23). The same activities at follow-up are reported for 12, 13, and 12 patients, respectively. Data could not be analyzed during therapy for Wii-golf for one patient (with moderate motor-function) due to tonic muscle activity and during Wii-tennis for another patient (low motor-function) with limited shoulder movement that prevented this activity. No adverse events were reported, either minor or major.

### Changes in EMG as a Consequence of Therapy (Early to Late Therapy)

**Figure 3:**
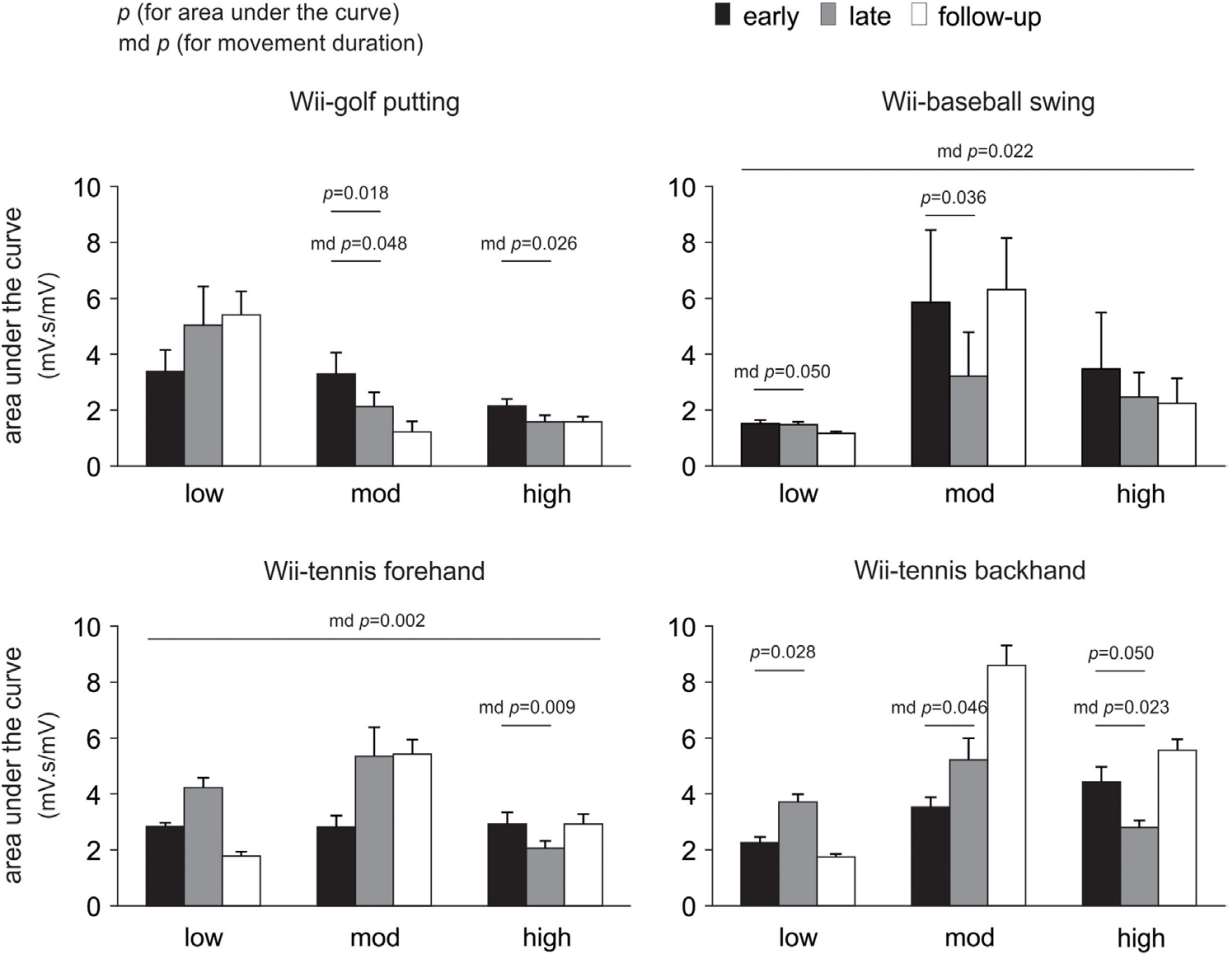
Pooled data showing changes in electromyography over time. Changes are presented as mean ± SE for the area under the curve according to the level of poststroke motor-function. Significant changes in movement duration (md) are also indicated.

Changes in the area under the curve, movement duration, and peak amplitude of muscle activation from early to late therapy for different Wii-activities are listed in Table 2 and illustrated in Figure 3. For Wii-golf putting, the movement duration decreased significantly for patients with moderate (*p* = 0.048) and high motor-function levels (*p* = 0.026); area under the curve was also reduced for patients with moderate motor-function (*p* = 0.018). The movement duration of Wii-baseball swings showed a trend toward decreasing for patients with low motor-function (*p* = 0.050), while there was a reduction in the area under the curve for the patients with moderate motor-function (*p* = 0.036). The movement duration of Wii-tennis forehand (*p* = 0.009) and backhand (*p* = 0.023) decreased for patients with high motor-function by late therapy. Moreover, the movement duration of Wii-tennis backhand decreased (*p* = 0.046) for patients with moderate motor-function. The area under the curve (*p* = 0.028) and peak amplitude (*p* = 0.043) increased for patients with low motor-function during backhand swings, while the area under the curve (*p* = 0.050) showed a reduction trend for patients with high motor-function.

**Table 2:** Therapy-induced electromyography changes from early to late therapy for patients with low, moderate, and high motor-function levels.

	Low early	Low late	*p* Value	Mod early	Mod late	*p* Value	High early	High late	*p* Value
**Wii-golf putting**	***n* = 8**	***n* = 8**		***n* = 7**	***n* = 7**		***n* = 8**	***n* = 8**	
Area under the curve (mV.s/mV; median, IQR)	2.24 (1.76–4.96)	3.26 (2.05–7.27)	0.401	2.36 (1.80–7.00)	1.75 (0.64–2.02)	**0.018**	2.13 (1.35–3.12)	1.48 (1.01–1.86)	0.161
Movement duration (s; mean ± SE)	3.32 ± 0.39	3.83 ± 1.02	0.600	3.08 ± 0.47	2.18 ± 0.47	**0.048**	2.23 ± 0.26	1.89 ± 0.19	**0.026**
Peak amplitude (mV/mV; median, IQR)	1.53 (1.21–3.86)	2.00 (1.21–7.09)	0.674	1.77 (1.31–3.57)	1.98 (1.29–2.23)	0.612	2.28 (1.31–3.08)	1.71 (1.31–2.68)	0.327
**Wii-baseball swing**	***n* = 8**	***n* = 8**		***n* = 8**	***n* = 8**		***n* = 8**	***n* = 8**	
Area under the curve (mV.s/mV; median, IQR)	1.41 (1.33–1.76)	1.40 (1.30–1.72)	1.000	3.09 (1.30–8.90)	1.85 (0.90–3.26)	**0.036**	1.87 (0.84–4.78)	2.11 (0.49–4.16)	0.123
Movement duration (s; mean ± SE)	0.78 (0.64–1.08)	0.69 (0.57–0.82)	*0.050*	1.31 ± 0.26	1.01 ± 0.17	*0.095*	0.75 ± 0.15	0.73 ± 0.11	0.662
Peak amplitude (mV/mV; median, IQR)	1.49 (1.40–1.55)	1.36 (1.28–1.55)	0.263	6.17 (1.87–12.39)	4.77 (2.17–12.18)	0.575	5.46 (3.58–15.58)	5.72 (2.20–12.44)	0.123
**Wii-tennis forehand**	***n* = 7**	***n* = 7**		***n* = 8**	***n* = 8**		***n* = 8**	***n* = 8**	
Area under the curve (mV.s/mV; median, IQR)	2.72 (2.20–3.57)	3.95 (2.51–5.02)	*0.091*	2.57 (1.92–4.20)	3.51 (2.11–5.13)	*0.093*	2.10 (1.48–3.82)	1.96 (1.27–3.00)	0.123
Movement duration (s; mean ± SE)	1.81 ± 0.25	1.90 ± 0.34	0.869	1.85 ± 0.18	1.94 ± 0.20	0.682	2.03 ± 0.23	1.55 ± 0.20	**0.009**
Peak amplitude (mV/mV; median, IQR)	3.90 (2.27–5.42)	4.10 (3.04–6.56)	0.128	2.49 (1.69–4.48)	4.16 (2.90–9.24)	*0.093*	2.75 (2.13–5.83)	3.01 (1.74–7.21)	0.401
**Wii-tennis backhand**	***n* = 7**	***n* = 7**		***n* = 8**	***n* = 8**		***n* = 8**	***n* = 8**	
Area under the curve (mV.s/mV; median, IQR)	1.71 (1.40–3.12)	3.32 (2.30–5.19)	**0.028**	3.33 (1.82–5.39)	3.41 (1.54–5.71)	0.575	3.30 (2.21–6.07)	2.62 (2.06–3.78)	*0.050*
Movement duration (s; mean ± SE)	1.54 ± 0.25	1.65 ± 0.19	0.862	1.87 ± 0.15	1.64 ± 0.09	**0.046**	1.76 ± 0.13	1.48 ± 0.15	**0.023**
Peak amplitude (mV/mV; median, IQR)	2.57 (1.75–5.33)	4.27 (2.75–7.13)	**0.043**	3.75 (3.00–5.38)	5.29 (4.02–10.12)	*0.069*	4.47 (4.00–13.21)	4.92 (3.37–5.67)	0.123

*Significant changes are highlighted in bold, and trends in italics*.

*Mod, moderate; early, early therapy; late, late therapy; IQR, interquartile range*.

### Changes in EMG over Time (Early Therapy, Late Therapy, and Follow-up)

Linear mixed models demonstrated an effect of motor-function during Wii-golf (*p* = 0.009) and an interaction between time and motor-function (*p* = 0.016) for movement duration. Wii-baseball movement duration changed as a function of time (*p* = 0.022). Wii-tennis forehand swing movement duration demonstrated an effect of time (*p* = 0.002) and an interaction of time and motor-function (*p* = 0.005). In addition, the pattern of change for Wii-tennis backhand movement duration over time showed a non-significant trend (*p* = 0.059). For Wii-golf area under the curve, there was an effect of the level of motor-function (*p* = 0.034), with a non-significant trend for an interaction between time and motor-function (*p* = 0.072). Finally, there was a non-significant trend for the effect of time on Wii-baseball area under the curve (*p* = 0.070).

### Changes in Clinical Assessments with Therapy (Pre- to Post-Therapy)

**Figure 4:**
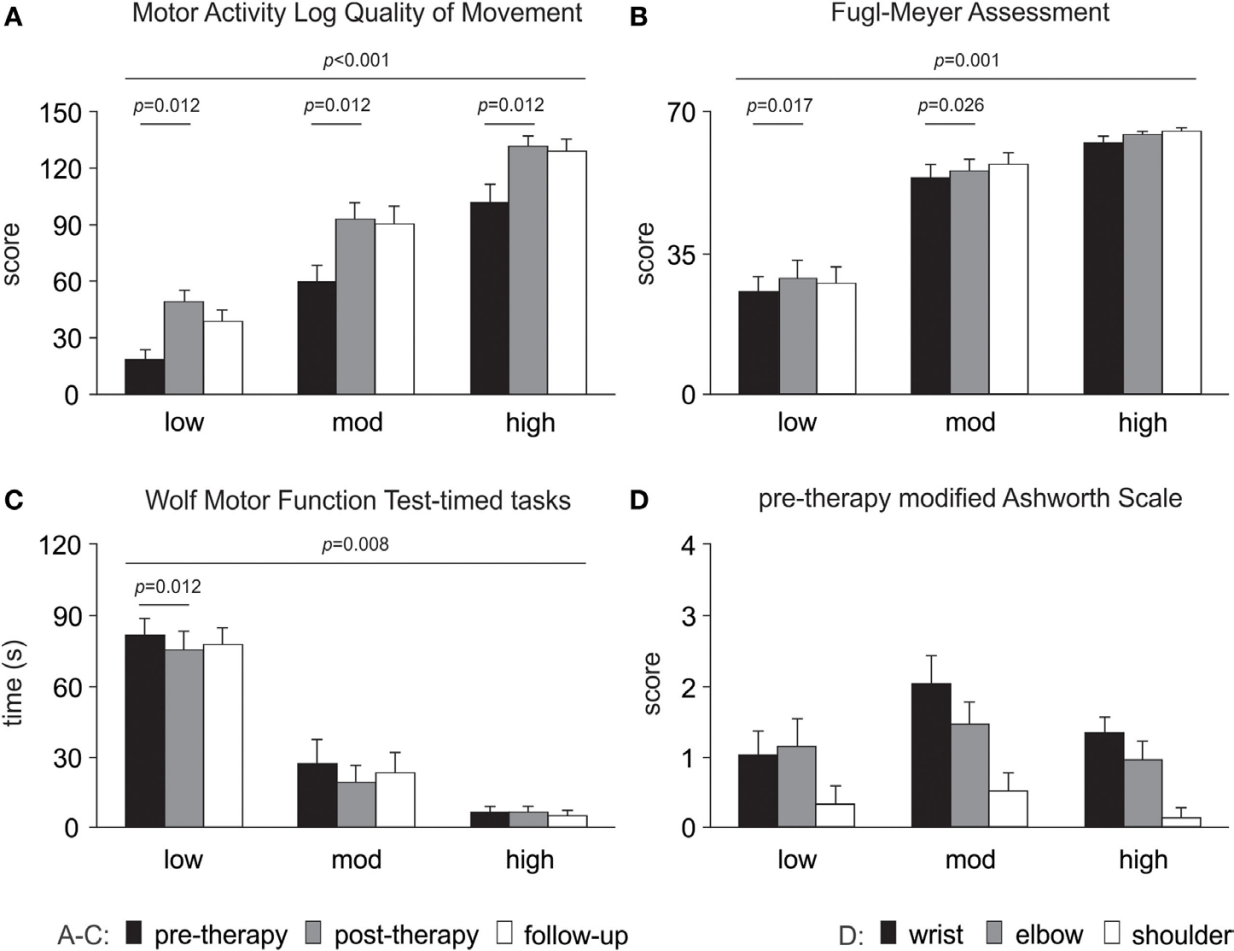
Changes in clinical assessments over time. Significant changes are evident for **(A)** Motor Activity Log Quality of Movement scale, **(B)** upper limb motor Fugl-Meyer Assessment and **(C)** Wolf Motor Function Test-timed tasks (note a decrease in time reflects improved performance). **(D)** Modified Ashworth Scale pre-therapy (baseline) data are presented as there were no changes over time. All data are presented as mean ± SE.

Clinical assessment data showed improvements from pre- to post-therapy for the pooled data (*n* = 24) for WMFT timed tasks (*p* = 0.004), FMA (*p* = 0.001) and MALQOM (*p* < 0.001). There were no changes in Ashworth scores at wrist (*p* = 0.355), elbow (*p* = 0.796), or shoulder (*p* = 0.592) at post-therapy. The detailed results for each level of motor-function are presented in Figure 4.

### Changes in Clinical Assessments over Time (Pre-Therapy, Post-Therapy, and Follow-up)

Linear mixed models demonstrated improvement over time for WMFT-tt (*p* = 0.008), FMA (*p* = 0.001), and MALQOM (*p* < 0.001). There was an effect of the level of motor-function for the WMFT-tt, FMA, and MALQOM (*p* < 0.001 for all).

### Game Performance

Game performance improved from early to late therapy. The successful Wii-golf swings (landing the ball in the hole) increased by 30.7% (*p* = 0.004). The number of Wii-baseball hits increased by 51.5% (*p* < 0.001), while the combined Wii-tennis forehand and backhand hits increased by 91.5% (*p* = 0.012). The increase in game scores was sustained at 6-month follow-up with significant improvements over time for Wii-golf (*p* = 0.004), -baseball (*p* < 0.001), and -tennis (*p* = 0.022).

### Qualitative EMG Observations

As detailed earlier, EMG data demonstrated therapy-induced changes particularly for patients with low motor-function. Qualitative observations included that the EMG signal from muscles with prolific single motor unit activity became more compound by late therapy indicating increased motor unit recruitment; tonic activity became more phasic; and there were more distinct and task-related bursts of EMG in more muscles (see Figure 2B).

### Relationship between Changes in EMG and Clinical Assessments

The correlation between therapy-induced changes in clinical motor assessments (WMFT-tt, FMA, and MALQOM) and the changes in EMG parameters (of Wii-golf, -baseball, -forehand, and -backhand) were examined. For the pooled data, there was no relationship between the change in the clinical assessments and the change in area under the curve of each activity (*r* < 0.39, *p* > 0.18 for all comparisons) and the change in movement duration of the same activities (*r* < 0.20, *p* > 0.90 for all comparisons). Furthermore, within each level of motor-function, no relationship was found either for area under the curve (low: *r* < 0.45, *p* > 0.78; moderate: *r* < 0.71, *p* > 0.14; high: *r* < 0.54, *p* > 0.50), or movement duration (low: *r* < 0.73, *p* > 0.12; moderate: *r* < 0.62, *p* > 0.30), with the single exception of a significant correlation that was found between the change in Wii-baseball movement duration and clinical assessments in patients with high motor-function (*r* = −0.81, *p* = 0.048).

## Discussion

**Figure 5:**
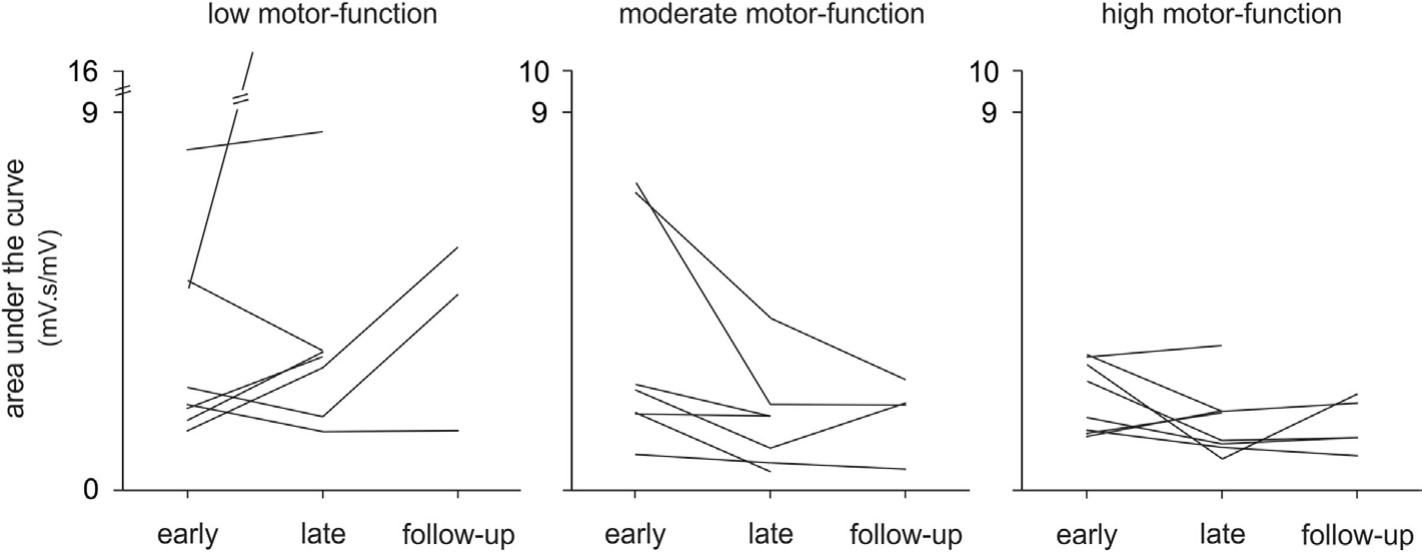
Changes in electromyography over time, individual patient data. The mean area under the curve is shown for each patient (*n* = 24) with low, moderate, and high motor-function levels at early and late therapy and for a subset of patients (*n* = 13) at 6-month follow-up during Wii-golf putting.

In this study, we investigated changes in upper limb muscle activation to gain greater insight into the neurophysiological mechanisms of improved motor-function in a heterogeneous stroke cohort from early to late therapy and with a subset of patients at 6-month follow-up. To the best of our knowledge, this study is the first longitudinal analysis of upper limb EMG *during* therapy in chronic stroke. Moreover, the movements we studied were largely unconstrained in time and space with no experimentally pre-defined start- or end-points. Although all patients made significant improvements on clinical assessments, and muscle activation changed with therapy, contrary to our hypothesis, there was no consistent pattern of change in EMG in the pooled data or within any motor-function group (see Figure 5). Qualitatively, there were more discrete bursts of EMG, less tonic activity, and less co-contraction (see Figure 2B). These findings demonstrate that there is no one pattern of improvement regardless of the level of motor-function, that task-related EMG demands may increase or decrease, and that both are associated with improved therapy (game) performance and independence in everyday tasks.

One of the two primary objectives of Wii-based Movement Therapy is movement quality, rather than EMG activity or game scores. Thus, therapy activities were not modified in any way to enhance the recorded EMG signals (25, 48, 51). Wii-activities provide a wide range of movement demands. For example, the self-paced Wii-golf putting requires movements that are smaller and more-controlled compared to the externally-timed Wii-baseball and -tennis swings that can be used to target movement speed, range, power, and coordination (58).

The pattern of change in the EMG signals by late therapy was complex and variable. There were distinct differences between patients with low motor-function and those with moderate and high motor-function levels (49, 59, 60). The pattern of change in the area under the curve showed an increase for patients with low motor-function from early to late therapy for all activities except Wii-baseball in which there was no change. This pattern was reversed for patients with high motor-function, while those with moderate motor-function had a reduction in Wii-golf putting and -baseball swings and an increase in Wii-tennis forehand and backhand hits. In contrast, clinical motor assessments with the exception of the modified Ashworth Scale improved for the pooled cohort. The differences between the levels of motor-function arise as a consequence of the limitation of each assessment tool, particularly for the modified Ashworth Scale (46, 47, 61). These data emphasize differences in the level of residual voluntary motor capacity in chronic stroke. Although patients with low motor-function retain the capacity to improve, the level of impairment may limit their ability to participate fully in each activity and hence circumscribe the potential for improvement. Those with moderate motor-function have the greatest capacity for improvement, whereas patients with high motor-function are the most likely to show improvements (34). Regardless of changes in EMG and movement ability, the MALQOM data demonstrate that every patient became more independent in activities of daily living (46, 62).

Muscle activation after stroke is typically analyzed during simple single-joint tasks (20, 22, 43), or during experimentally constrained movements (41, 45, 63). Our analysis focused on the area under the curve because it is the product of EMG amplitude and the movement duration and so provides a more holistic view of the movement. While changes in movement duration were larger, there were fewer changes in peak amplitude. In this study, the movements were largely unconstrained, in that patients chose their own starting position within a task-dependent framework and the end position predominantly reflected movement capacity. When essential, positioning assistance was provided either with the less-affected arm or by the therapist. Wii-golf was self-paced, Wii-baseball was paced by the game but required the development of fine response timing, while the timing of Wii-tennis was variable from swing to swing. Thus, these activities provide a better reflection of upper limb use in everyday life than more constrained experimental tasks and are more likely to reflect the neurophysiology underlying functionally relevant changes in poststroke motor-function.

Despite monotonic improvements in clinical assessments, there were heterogeneous changes in muscle activation patterns during therapy. Patients in each level of motor-function used different strategies to perform a task according to their specific neuromuscular limitations. Wii-activities target various muscles and movements (58), and differences in residual voluntary muscle activation alter the goals of therapy for each patient and result in movement patterns that differ from those of healthy control subjects (46, 50). These differences precluded analysis based on a single pre-determined muscle for all patients. Single muscle analysis provides some information about corticomotor changes in motor control but little about the coordination of muscles in the production of a complex movement. For this reason, these data were further analyzed to investigate the neuromuscular coordination of these complex movements [see (64)].

### Clinical Implications

Although all patients completed a standardized protocol of Wii-based Movement Therapy and improved on clinical assessments, the pattern of change in EMG differed. Each motor-function group had a dominant pattern; yet, there was a large amount of variation within each group (see Figure 5). Regardless of the pattern of improvement in EMG, therapy-induced changes were reflected in improvements in independence (MALQOM) and quality of movement in activities of daily living that were unrelated to the content of therapy (see Figure 4). These data suggest that Wii-based Movement Therapy tasks can target different aspects of motor control. For example, the area under the curve in Wii-golf putting increased over time for patients with low motor-function showing that these patients were able to perform longer and stronger movements after therapy. Therefore, Wii-golf can be used to focus on slower and more-controlled movements with a sustained position at the end of the swing to target positional stability. Patients with moderate and high motor-function levels had a reduction in area under the curve over time indicating more coordinated and smaller movements, confirmed by video recordings. The increased area under the curve during Wii-tennis for patients with low and moderate motor-function levels suggests a greater ability to move from one side of the body to the other so that fuller “strokes” were played for both forehand and backhand as seen in video recordings. In contrast, the reduced area under the curve for patients with high motor-function suggests more efficient movements that can be used to target the difference between making one movement and preparing for the next movement. An ancillary benefit of these changes is that Wii-tennis also promotes greater stepping (25), which we hypothesize will also improve standing balance.

Game scores indicated that patients improved in terms of game performance; however, game scores do not necessarily reflect performance. Task difficulty is increased during therapy based on patient progress (25, 51). The increased difficulty typically lowers game scores resulting in a pattern of oscillating scores with a trend of improvement over time.

Electromyography analysis provides a mean of demonstrating neurophysiological changes in chronic stroke underlying significant improvements in clinical assessments, suggesting that ongoing rehabilitation is effective. Preliminary data from our group (65) emphasize that a continuous trajectory of improvement in clinical measures is possible when additional periods of therapy are provided in the chronic phase of stroke.

### Study Limitations

In this study, the sample size within each level of motor-function is small. Yet, the pooled sample size is comparable to published studies of poststroke EMG (66–69). A wide range of poststroke motor impairment is included in this study based on clinical assessment data. Patients with low motor-function are not typically recruited in most neurophysiological studies due to methodological challenges. These patients need special care during assessment and therapy sessions due to pain, limited mobility and fatigue. Significant upper limb motor heterogeneity, together with the largely unconstrained movements during therapy and EMG acquisition from a variety of muscles increase the complexity of EMG analysis and interpretation.

The absence of healthy control subjects is a limitation to this study. Given that therapy focuses on the quality of movements and increasing the use of more-affected upper limb in everyday activities, the instructions given to stroke patients are different from those given to healthy subjects performing the same Wii-activities. The movements used in therapy are designed to replicate movement patterns of real-world sporting activities, despite all activity being targeted as far as possible, to use of the more-affected upper limb alone. Initially movements may be fractionated into their constituent parts for practice using the principles of shaping (70). By the end of therapy, most patients are able to perform the necessary reconstituted whole movements with game performance and scores used only as a motivational tool (46). In contrast, healthy subjects playing the same games used more modified movements than the real-world equivalent, and despite skill acquisition during Wii-Sports, showed no change in clinical assessments of motor-function (50). Thus, these differences in movement patterns may limit the utility of comparisons between healthy subjects and patients.

Although patients were drawn from several concurrent Wii-based Movement Therapy studies, all received the standardized protocol and we have seen very little variation in therapy outcomes across trials (46, 50, 62). The patients were instructed to perform the same task-dependent movements; however, the variability in movement strategies, tonic muscle activity, level of impairment, and the ability to voluntarily relax muscles after each movement prevented the use of single muscle for all analyses. This approach adds complexity to the analysis but was preferable to attempting to ensure all patients activated a given muscle as this was either not possible for some patients or would have produced extraneous or counterproductive movements. To enable comparisons between patients and between multiple time-points, EMG data were normalized to a standardized condition, even though it has been suggested that EMG normalization in poststroke data might lead to higher variability (45). An unsupported weighted condition was used for normalization as this was considered more reliable for stroke survivors than a maximal voluntary contraction that might be restricted due to pain (71). Such variability is an inherent problem of longitudinal poststroke therapy studies when the aim of therapy is to change the muscles themselves, in addition to changing the neuromuscular control of those muscles.

## Conclusion

This study demonstrates the magnitude of the variability in poststroke response to therapy and Wii-based Movement Therapy-induced changes in upper limb EMG in chronic stroke. The absence of correlations between EMG activity, game performance, and clinical assessments highlights the complexity and heterogeneity that are characteristic of stroke, even in the chronic period. Despite the absence of readily identifiable patterns across the pooled EMG data, the pattern of changes in EMG was associated with the level of residual voluntary motor capacity. The heterogeneity within each level shown in this EMG study was not evident using clinical assessments of motor-function, although improved independence in everyday activities was evident for all patients. These data emphasize the importance of examining individual patient responses to therapy using multiple tools, as rehabilitation efficacy will be underestimated when data are pooled.

## Ethics Statement

This study was carried out in accordance with the recommendations of St. Vincent Hospital Human Research Ethics Committee, Sydney with written consent from all patients. All patients gave written informed consent in accordance with the Declaration of Helsinki. The protocol was approved by St. Vincent Hospital Human Research Ethics Committee.

## Author Contributions

PMcN conceived, designed, and supervised the study and manuscript preparation. NH-S assisted with data collection, developed multiple code scripts, analyzed data, and drafted the manuscript. TT implemented data collection. AT-B implemented therapy. CS undertook clinical assessments. All authors contributed to manuscript revision.

## Conflict of Interest Statement

The authors declare that the research was conducted in the absence of any commercial or financial relationships that could be construed as a potential conflict of interest.
